# 4-(Dimethyl­amino)benzaldehyde

**DOI:** 10.1107/S160053680801581X

**Published:** 2008-06-07

**Authors:** Bo Gao, Jian-Liang Zhu

**Affiliations:** a Marine College, Zhejiang Institute of Communications, Hangzhou 311112, People’s Republic of China

## Abstract

The title compound, C_9_H_11_NO, crystallizes with two independent but essentially identical mol­ecules in the asymmetric unit, which are linked *via* a C—H⋯π inter­action. In both mol­ecules, the aldehyde and dimethyl­amine groups are essentially coplanar with the attached benzene ring. In the crystal structure, C—H⋯O hydrogen bonds link one type of independent mol­ecules into a chain along the *a* axis. In addition, the structure is stabilized by π–π stacking inter­actions involving the benzene rings [centroid-to-centroid distance = 3.697 (2) Å].

## Related literature

For synthesis, see: Wu & Zhou (2005 ▶). For general background, see: Kawski *et al.* (2007 ▶). For related structures, see: Reffner & McCrone (1959 ▶); Dattagupta & Saha (1973 ▶); Herbstein *et al.* (1984 ▶); Mahadevan *et al.* (1982 ▶); Habibi *et al.* (2007 ▶).
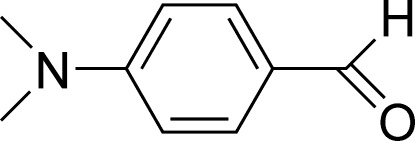


## Experimental

### 

#### Crystal data

C_9_H_11_NO
*M* *_r_* = 149.19
Monoclinic, 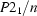

*a* = 10.356 (6) Å
*b* = 7.686 (4) Å
*c* = 20.8434 (13) Å
β = 96.808 (13)°
*V* = 1647.4 (12) Å^3^
*Z* = 8
Mo *K*α radiation
μ = 0.08 mm^−1^
*T* = 123 (2) K
0.27 × 0.23 × 0.20 mm

#### Data collection

Bruker SMART CCD area-detector diffractometer
Absorption correction: multi-scan (*SADABS*; Bruker, 2002 ▶) *T*_min_ = 0.979, *T*_max_ = 0.981
9835 measured reflections
2869 independent reflections
1826 reflections with *I* > 2σ(*I*)
*R*_int_ = 0.058

#### Refinement

*R*[*F* ^2^ > 2σ(*F* ^2^)] = 0.057
*wR*(*F* ^2^) = 0.160
*S* = 1.01
2869 reflections
199 parameters
H-atom parameters constrained
Δρ_max_ = 0.22 e Å^−3^
Δρ_min_ = −0.31 e Å^−3^

### 

Data collection: *SMART* (Bruker, 2002 ▶); cell refinement: *SAINT* (Bruker, 2002 ▶); data reduction: *SAINT*; program(s) used to solve structure: *SHELXS97* (Sheldrick, 2008 ▶); program(s) used to refine structure: *SHELXL97* (Sheldrick, 2008 ▶); molecular graphics: *SHELXTL* (Sheldrick, 2008 ▶); software used to prepare material for publication: *SHELXTL*.

## Supplementary Material

Crystal structure: contains datablocks I, global. DOI: 10.1107/S160053680801581X/ci2602sup1.cif

Structure factors: contains datablocks I. DOI: 10.1107/S160053680801581X/ci2602Isup2.hkl

Additional supplementary materials:  crystallographic information; 3D view; checkCIF report

## Figures and Tables

**Table 1:** Hydrogen-bond geometry (Å, °) *Cg*1 is the centroid of the C11–C16 ring.

*D*—H⋯*A*	*D*—H	H⋯*A*	*D*⋯*A*	*D*—H⋯*A*
C9—H9*A*⋯O1^i^	0.96	2.57	3.459 (3)	155
C3—H3⋯*Cg*1	0.93	2.78	3.593 (3)	146

Symmetry code: (i) 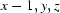.

**Fig. 1.**
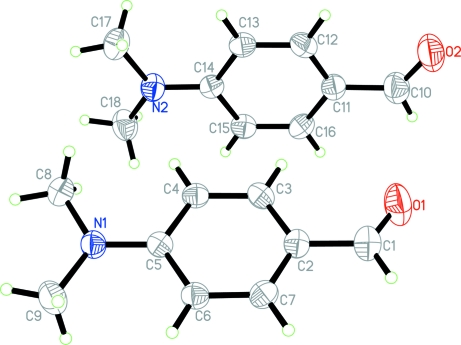
The asymmetric unit of the title compound. Displacement ellipsoids are drawn at the 30% probability level.

## supplementary crystallographic information

### Comment

4-Dimethylaminobenzaldehyde (DMABA) is an important intermediate of dyes and medicine. It belongs to the same family as 4-(dimethylamino)benzonitrile (DMABN) which exhibits dual fluorescence and was a subject of extensive investigations (Kawski *et al.*, 2007). Although the unit-cell parameters of DMABA have been reported (Reffner & McCrone, 1959), to our knowledge there is no report on the crystal structure of DMABA. The crystal structures of DMABA hydrobromide (Dattagupta & Saha, 1973), a 1:1 complex in which DMABA acts as a guest molecule in channels (Herbstein *et al.*, 1984), a tin complex in which DMABA serves as a ligand coordinating through its O atom (Mahadevan *et al.*, 1982), and of a 1:1 cocrystal of DMABA and 6-phenyl-1,3,5-triazine-2,4-diamine (Habibi *et al.*, 2007) have been reported. We report here the crystal structure of the title compound.

The title compound crystallizes with two independent but essentially identical molecules in the asymmetric unit (Fig. 1). In both molecules, the aldehyde and dimethylamino groups are essentially coplanar with the attached benzene ring, similar to those observed in above crystal structures. The mean planes through the non-hydrogen atoms of two independent molecules form a dihedral angle of 76.42 (5)°. The two independent molecules are linked via a C—H···π interaction involving the C3—H3 group and C11–C16 benzene ring (Table 1).

In the crystal structure, C—H···O hydrogen bonds (Table 1) link one type of independent molecules into a chain along the *a* axis. In addition, the structure is stabilized by stacking interactions between the inversion related C11–C16 benzene rings [centroid–centroid distance is 3.697 (2) Å].

### Experimental

The title compound was prepared according to the literature method (Wu & Zhou, 2005). Crystals suitable for X-ray analysis were obtained by slow evaporation of a isoproanol solution at room temperature (m.p. 343–347 K).

### Refinement

H atoms were positioned geometrically (C—H = 0.93–0.96 Å) and refined using a riding model, with *U*_iso_(H) = 1.2–1.5*U*_eq_(C).

### Crystal data

**Table:**

C_9_H_11_NO	*F*(000) = 640
*M**_r_* = 149.19	*D*_x_ = 1.203 Mg m^−^^3^
Monoclinic, *P*2_1_/*n*	Mo *K*α radiation, λ = 0.71073 Å
Hall symbol: -P 2yn	Cell parameters from 2869 reflections
*a* = 10.356 (6) Å	θ = 2–25.0°
*b* = 7.686 (4) Å	µ = 0.08 mm^−^^1^
*c* = 20.8434 (13) Å	*T* = 123 K
β = 96.808 (13)°	Block, colourless
*V* = 1647.4 (12) Å^3^	0.27 × 0.23 × 0.20 mm
*Z* = 8	

### Data collection

**Table:**

Bruker SMART CCD area-detector diffractometer	2869 independent reflections
Radiation source: fine-focus sealed tube	1826 reflections with *I* > 2σ(*I*)
graphite	*R*_int_ = 0.058
φ and ω scans	θ_max_ = 25.0°, θ_min_ = 2.0°
Absorption correction: multi-scan (*SADABS*; Bruker, 2002)	*h* = −12→12
*T*_min_ = 0.979, *T*_max_ = 0.981	*k* = −9→9
9835 measured reflections	*l* = −22→24

### Refinement

**Table:**

Refinement on *F*^2^	Primary atom site location: structure-invariant direct methods
Least-squares matrix: full	Secondary atom site location: difference Fourier map
*R*[*F*^2^ > 2σ(*F*^2^)] = 0.057	Hydrogen site location: inferred from neighbouring sites
*wR*(*F*^2^) = 0.160	H-atom parameters constrained
*S* = 1.01	*w* = 1/[σ^2^(*F*_o_^2^) + (0.0934*P*)^2^] where *P* = (*F*_o_^2^ + 2*F*_c_^2^)/3
2869 reflections	(Δ/σ)_max_ = 0.001
199 parameters	Δρ_max_ = 0.22 e Å^−^^3^
0 restraints	Δρ_min_ = −0.31 e Å^−^^3^

### Special details

**Table:**

Geometry. All e.s.d.'s (except the e.s.d. in the dihedral angle between two l.s. planes) are estimated using the full covariance matrix. The cell e.s.d.'s are taken into account individually in the estimation of e.s.d.'s in distances, angles and torsion angles; correlations between e.s.d.'s in cell parameters are only used when they are defined by crystal symmetry. An approximate (isotropic) treatment of cell e.s.d.'s is used for estimating e.s.d.'s involving l.s. planes.
Refinement. Refinement of *F*^2^ against ALL reflections. The weighted *R*-factor *wR* and goodness of fit *S* are based on *F*^2^, conventional *R*-factors *R* are based on *F*, with *F* set to zero for negative *F*^2^. The threshold expression of *F*^2^ > σ(*F*^2^) is used only for calculating *R*-factors(gt) *etc*. and is not relevant to the choice of reflections for refinement. *R*-factors based on *F*^2^ are statistically about twice as large as those based on *F*, and *R*- factors based on ALL data will be even larger.

### Fractional atomic coordinates and isotropic or equivalent isotropic displacement parameters (Å^2^)

**Table:**

	*x*	*y*	*z*	*U*_iso_*/*U*_eq_	
O1	1.26881 (13)	0.5751 (2)	0.18550 (9)	0.1025 (6)	
O2	1.37916 (14)	0.2647 (3)	0.04689 (9)	0.1088 (6)	
C9	0.57238 (17)	0.7676 (3)	0.21132 (10)	0.0742 (6)	
H9A	0.4815	0.7498	0.1976	0.111*	
H9B	0.5939	0.8876	0.2055	0.111*	
H9C	0.5912	0.7373	0.2561	0.111*	
C8	0.57965 (16)	0.5577 (3)	0.12110 (9)	0.0653 (5)	
H8A	0.4876	0.5723	0.1213	0.098*	
H8B	0.6017	0.4370	0.1271	0.098*	
H8C	0.6040	0.5968	0.0805	0.098*	
C18	0.69062 (19)	0.0550 (3)	0.08262 (11)	0.0809 (6)	
H18A	0.5991	0.0674	0.0694	0.121*	
H18B	0.7102	0.0920	0.1267	0.121*	
H18C	0.7151	−0.0647	0.0789	0.121*	
C17	0.68913 (18)	0.2440 (3)	−0.01380 (10)	0.0776 (6)	
H17A	0.5978	0.2267	−0.0120	0.116*	
H17B	0.7132	0.1938	−0.0528	0.116*	
H17C	0.7079	0.3664	−0.0132	0.116*	
C5	0.78138 (15)	0.66025 (19)	0.18319 (8)	0.0453 (4)	
C2	1.05546 (16)	0.6610 (2)	0.20311 (9)	0.0524 (5)	
N1	0.64859 (12)	0.65921 (18)	0.17319 (7)	0.0542 (4)	
C4	0.85497 (16)	0.5535 (2)	0.14644 (8)	0.0508 (4)	
H4	0.8126	0.4817	0.1147	0.061*	
C3	0.98899 (16)	0.5538 (2)	0.15671 (8)	0.0528 (5)	
H3	1.0356	0.4810	0.1322	0.063*	
C6	0.84982 (16)	0.7670 (2)	0.23045 (8)	0.0529 (5)	
H6	0.8042	0.8385	0.2559	0.063*	
C7	0.98356 (16)	0.7666 (2)	0.23942 (8)	0.0559 (5)	
H7	1.0268	0.8391	0.2706	0.067*	
C1	1.19714 (19)	0.6632 (3)	0.21338 (11)	0.0716 (6)	
H1	1.2358	0.7399	0.2444	0.086*	
C14	0.89524 (16)	0.1659 (2)	0.05031 (8)	0.0500 (4)	
N2	0.76244 (14)	0.1612 (2)	0.04153 (8)	0.0629 (5)	
C13	0.96580 (17)	0.2560 (2)	0.00770 (8)	0.0567 (5)	
H13	0.9217	0.3114	−0.0281	0.068*	
C11	1.16899 (17)	0.1858 (2)	0.07077 (10)	0.0583 (5)	
C15	0.96619 (17)	0.0836 (2)	0.10370 (9)	0.0581 (5)	
H15	0.9226	0.0210	0.1327	0.070*	
C16	1.09953 (17)	0.0953 (2)	0.11309 (9)	0.0609 (5)	
H16	1.1446	0.0410	0.1488	0.073*	
C12	1.09922 (18)	0.2640 (2)	0.01775 (9)	0.0612 (5)	
H12	1.1437	0.3234	−0.0117	0.073*	
C10	1.3101 (2)	0.1939 (3)	0.08206 (12)	0.0812 (6)	
H10	1.3506	0.1406	0.1192	0.097*	

### Atomic displacement parameters (Å^2^)

**Table:**

	*U*^11^	*U*^22^	*U*^33^	*U*^12^	*U*^13^	*U*^23^
O1	0.0548 (8)	0.1082 (13)	0.1464 (15)	0.0050 (8)	0.0195 (9)	−0.0127 (11)
O2	0.0684 (10)	0.1282 (15)	0.1327 (15)	−0.0202 (9)	0.0242 (10)	−0.0258 (12)
C9	0.0562 (11)	0.0765 (14)	0.0926 (15)	−0.0019 (10)	0.0201 (11)	−0.0170 (12)
C8	0.0548 (10)	0.0758 (13)	0.0633 (12)	−0.0026 (9)	−0.0012 (9)	−0.0029 (10)
C18	0.0624 (12)	0.0841 (15)	0.0979 (16)	−0.0089 (11)	0.0168 (11)	0.0110 (13)
C17	0.0652 (12)	0.0777 (15)	0.0868 (15)	0.0094 (11)	−0.0045 (11)	0.0036 (12)
C5	0.0497 (10)	0.0397 (9)	0.0472 (10)	−0.0017 (7)	0.0080 (8)	0.0049 (7)
C2	0.0497 (10)	0.0492 (10)	0.0580 (11)	−0.0017 (8)	0.0049 (8)	0.0099 (8)
N1	0.0462 (8)	0.0562 (9)	0.0601 (9)	−0.0013 (6)	0.0062 (7)	−0.0077 (7)
C4	0.0564 (10)	0.0476 (10)	0.0486 (10)	−0.0012 (8)	0.0065 (8)	−0.0038 (8)
C3	0.0554 (10)	0.0489 (10)	0.0562 (11)	0.0040 (8)	0.0159 (8)	0.0011 (8)
C6	0.0556 (10)	0.0487 (10)	0.0548 (11)	0.0015 (8)	0.0084 (8)	−0.0069 (9)
C7	0.0593 (11)	0.0507 (11)	0.0565 (11)	−0.0054 (8)	0.0019 (9)	−0.0047 (9)
C1	0.0550 (12)	0.0716 (14)	0.0876 (15)	−0.0007 (10)	0.0052 (11)	0.0053 (11)
C14	0.0566 (10)	0.0416 (9)	0.0516 (11)	0.0016 (8)	0.0052 (8)	−0.0036 (8)
N2	0.0534 (9)	0.0632 (10)	0.0714 (11)	0.0016 (7)	0.0046 (8)	0.0081 (8)
C13	0.0640 (11)	0.0527 (11)	0.0532 (11)	0.0016 (9)	0.0059 (9)	0.0043 (9)
C11	0.0556 (11)	0.0541 (11)	0.0653 (12)	−0.0026 (8)	0.0073 (9)	−0.0135 (9)
C15	0.0649 (12)	0.0516 (11)	0.0584 (11)	−0.0029 (9)	0.0098 (9)	0.0045 (9)
C16	0.0658 (12)	0.0564 (11)	0.0576 (11)	0.0019 (9)	−0.0045 (9)	0.0019 (9)
C12	0.0679 (12)	0.0585 (12)	0.0601 (12)	−0.0068 (9)	0.0189 (9)	−0.0022 (10)
C10	0.0660 (13)	0.0821 (15)	0.0966 (17)	−0.0098 (11)	0.0147 (12)	−0.0188 (13)

### Geometric parameters (Å, °)

**Table:**

O1—C1	1.204 (2)	C2—C3	1.389 (2)
O2—C10	1.212 (3)	C2—C1	1.457 (3)
C9—N1	1.448 (2)	C4—C3	1.379 (2)
C9—H9A	0.96	C4—H4	0.93
C9—H9B	0.96	C3—H3	0.93
C9—H9C	0.96	C6—C7	1.375 (2)
C8—N1	1.454 (2)	C6—H6	0.93
C8—H8A	0.96	C7—H7	0.93
C8—H8B	0.96	C1—H1	0.93
C8—H8C	0.96	C14—N2	1.366 (2)
C18—N2	1.450 (2)	C14—C13	1.399 (2)
C18—H18A	0.96	C14—C15	1.409 (2)
C18—H18B	0.96	C13—C12	1.374 (3)
C18—H18C	0.96	C13—H13	0.93
C17—N2	1.451 (2)	C11—C12	1.384 (3)
C17—H17A	0.96	C11—C16	1.389 (3)
C17—H17B	0.96	C11—C10	1.454 (3)
C17—H17C	0.96	C15—C16	1.374 (2)
C5—N1	1.366 (2)	C15—H15	0.93
C5—C4	1.407 (2)	C16—H16	0.93
C5—C6	1.407 (2)	C12—H12	0.93
C2—C7	1.386 (2)	C10—H10	0.93
			
N1—C9—H9A	109.5	C4—C3—C2	121.02 (16)
N1—C9—H9B	109.5	C4—C3—H3	119.5
H9A—C9—H9B	109.5	C2—C3—H3	119.5
N1—C9—H9C	109.5	C7—C6—C5	120.61 (16)
H9A—C9—H9C	109.5	C7—C6—H6	119.7
H9B—C9—H9C	109.5	C5—C6—H6	119.7
N1—C8—H8A	109.5	C6—C7—C2	121.65 (16)
N1—C8—H8B	109.5	C6—C7—H7	119.2
H8A—C8—H8B	109.5	C2—C7—H7	119.2
N1—C8—H8C	109.5	O1—C1—C2	126.2 (2)
H8A—C8—H8C	109.5	O1—C1—H1	116.9
H8B—C8—H8C	109.5	C2—C1—H1	116.9
N2—C18—H18A	109.5	N2—C14—C13	121.43 (16)
N2—C18—H18B	109.5	N2—C14—C15	121.09 (16)
H18A—C18—H18B	109.5	C13—C14—C15	117.46 (16)
N2—C18—H18C	109.5	C14—N2—C18	120.96 (15)
H18A—C18—H18C	109.5	C14—N2—C17	121.23 (15)
H18B—C18—H18C	109.5	C18—N2—C17	117.37 (15)
N2—C17—H17A	109.5	C12—C13—C14	121.13 (17)
N2—C17—H17B	109.5	C12—C13—H13	119.4
H17A—C17—H17B	109.5	C14—C13—H13	119.4
N2—C17—H17C	109.5	C12—C11—C16	117.64 (17)
H17A—C17—H17C	109.5	C12—C11—C10	122.03 (19)
H17B—C17—H17C	109.5	C16—C11—C10	120.32 (19)
N1—C5—C4	120.93 (15)	C16—C15—C14	120.27 (17)
N1—C5—C6	121.63 (15)	C16—C15—H15	119.9
C4—C5—C6	117.44 (15)	C14—C15—H15	119.9
C7—C2—C3	118.28 (16)	C15—C16—C11	121.97 (17)
C7—C2—C1	120.65 (17)	C15—C16—H16	119.0
C3—C2—C1	121.07 (17)	C11—C16—H16	119.0
C5—N1—C9	121.13 (15)	C13—C12—C11	121.50 (17)
C5—N1—C8	120.89 (14)	C13—C12—H12	119.3
C9—N1—C8	117.86 (14)	C11—C12—H12	119.3
C3—C4—C5	120.99 (16)	O2—C10—C11	125.1 (2)
C3—C4—H4	119.5	O2—C10—H10	117.4
C5—C4—H4	119.5	C11—C10—H10	117.4

### Hydrogen-bond geometry (Å, °)

**Table:**

*D*—H···*A*	*D*—H	H···*A*	*D*···*A*	*D*—H···*A*
C9—H9A···O1^i^	0.96	2.57	3.459 (3)	155
C3—H3···Cg1	0.93	2.78	3.593 (3)	146

Symmetry codes: (i) *x*−1, *y*, *z*.
